# Fine mapping QTL for resistance to VNN disease using a high-density linkage map in Asian seabass

**DOI:** 10.1038/srep32122

**Published:** 2016-08-24

**Authors:** Peng Liu, Le Wang, Sek-Man Wong, Gen Hua Yue

**Affiliations:** 1 Department of Biological Sciences, National University of Singapore, 14 Science Drive, Singapore 117543; 2 Temasek Life Sciences Laboratory, National University of Singapore, 1 Research Link, Singapore 117604; 3 National University of Singapore Suzhou Research Institute, Suzhou, Jiangsu, China 215123; 4 School of Biological Sciences, Nanyang Technological University, 6 Nanyang Drive, Singapore 637551

## Abstract

Asian seabass has suffered from viral nervous necrosis (VNN) disease. Our previous study has mapped quantitative trait loci (QTL) for resistance to VNN disease. To fine map these QTL and identify causative genes, we identified 6425 single nucleotide polymorphisms (SNPs) from 85 dead and 94 surviving individuals. Combined with 155 microsatellites, we constructed a genetic map consisting of 24 linkage groups (LGs) containing 3000 markers, with an average interval of 1.27 cM. We mapped one significant and three suggestive QTL with phenotypic variation explained (PVE) of 8.3 to 11.0%, two significant and two suggestive QTL with PVE of 7.8 to 10.9%, for resistance in three LGs and survival time in four LGs, respectively. Further analysis one QTL with the largest effect identified protocadherin alpha-C 2-like (*Pcdhac2*) as the possible candidate gene. Association study in 43 families with 1127 individuals revealed a 6 bp insertion-deletion was significantly associated with disease resistance. qRT-PCR showed the expression of *Pcdhac2* was significantly induced in the brain, muscle and skin after nervous necrosis virus (NNV) infection. Our results could facilitate marker-assisted selection (MAS) for resistance to NNV in Asian seabass and set up the basis for functional analysis of the potential causative gene for resistance.

The aquaculture industry has already provided more than 50% of seafood consumed worldwide^1^. The ever-growing demand for high-efficient aquaculture production to feed the world’s fast-growing population in the face of rapidly degenerating climate is still strong. To meet this challenge, selective breeding programs are adopted to accelerate the genetic gain and the subsequent performance, resulting in substantial improvement of production in several cultured aquatic species^2^. However, most traits of economic importance, including disease resistance are quantitative traits, controlled by quantitative trait loci (QTL) and influenced by environment^3^. Using traditional breeding methods to enhance genetic improvement has reached its bottleneck, due to slow speed, low efficiency, and being expensive for some traits like disease resistance^3^. Molecular breeding, including marker-assisted breeding (MAS) and a more advanced approach genomic selection (GS)^4^, possesses the great potential to overcome these difficulties. Those methods involve in using genetic markers in linkage disequilibrium (LD) with QTL or directly using quantitative trait nucleotide (QTN) to predict the phenotype and select the desirable individuals.

A large number of polymorphic genetic markers are essential for linkage map construction and QTL mapping. Currently, most linkage maps in aquatic species are built on genetic markers, such as restriction fragment length polymorphisms (RFLPs), amplified fragment polymorphisms (AFLPs) and simple sequence repeats (SSRs), and a few of them are constructed on single nucleotide polymorphisms (SNPs) or mixed types of markers^2^. To date, genetic maps of over 45 fish species have been reported^2^. However, development of these markers based on Sanger sequencing and genotyping large populations are slow, labor-intensive, time-consuming, and expensive. Moreover, the resolution of linkage maps built on these markers is moderate. Precise QTL mapping and further determination of causative polymorphisms, even positional cloning of the causative genes, require a great number of genome-wide polymorphic markers and high-resolution genetic maps to saturate the LD between marker and QTL^3^. It is hard for such conventional methods for marker development and genotyping to meet the increasing requirement for robust development of a large number of unbiased markers across the whole genome and high-density genotyping a large population at low cost^5^.

With the rapid advances of next-generation sequencing (NGS) technology, high-throughput sequencing-based marker discovery and genotyping have been quickly developed and adopted in many organisms^5^,^6^. The sequencing-based genotyping methods generally include multiplexing a proper number of samples with barcodes to greatly reduce per sample cost, and only sequencing a small fraction of a genome with increasing times of coverage, which naturally improves the accuracy of genotyping^5^. A small portion of a genome can be archived by reducing the complexity of the genome by restriction enzymes, such as the reduced-representation libraries (RRLs), restriction-site-associated DNA sequencing (RAD-seq) and genotyping-by-sequencing (GBS)^5^. Among them, GBS is a widely used approach especially in the non-model organisms without genome references or solid genomes^6^. The improved GBS approach employs two enzymes, one common cutter and one rare cutter. The combination of two enzyme cutters enables this approach to capture fragments associated with the rare cutters, which are roughly evenly distributed across the genome^6^. The hundreds of thousands fragments are then sequenced and millions of reads are produced, generating tens of thousands of unbiased SNPs spaced across the whole genomes. The feature of producing a larger amount of unbiased markers in an inexpensive way, enables GBS to become the preferable approach to build high-density and high-resolution maps, facilitating QTL mapping and genomic selection, even map-based cloning^6^. Riding the wave of GBS, genetic studies, high-density maps and/or QTL mapping for economically important traits have been conducted in several aquatic species. For example, GBS has been used in Atlantic salmon (*Salmo salar*) for disease resistance^7^ and genetic map^8^, blue catfish (*Ictalurus furcatus*) for genetic structure^9^, sea cucumber (*Apostichopus japonicas*) for body weight^10^, and Pacific white shrimp (*Litopenaeus vannamei*)^11^, Asian seabass (*Lates calcarifer*)^12^ and large yellow croaker (*Larimichthys crocea*)^13^ for growth traits. Besides these commercial fish species, GBS was also applied in several species of ecological importance, such as Chinook salmon (*Oncorhynchus tshawytscha*) for population structure^14^, sticklebacks (*Gasterosteus aculeatus*) for skeletal traits^15^ and Mexican tetra (*Astyanax mexicanus*) for genetic map^16^. These work demonstrated that GBS had the ability to construct high-density maps, which facilitate QTL mapping.

Asian seabass is an important food fish in Southeast Asia, with annual production reaching 75405 tons in 2012^17^. Despite the increasing economic importance of Asian seabass to this region, the genetic improvement of several important traits (e.g. disease resistance and meat quality) was lagging. Our lab has worked on Asian seabass for decades, focusing on growth^12^,^18^,^19^,^20^,^21^, meat quality^22^ and disease resistance^23^,^24^,^25^, through conventional and molecular breeding and selection^26^,^27^,^28^,^29^. The performance of these traits has been improved significantly since then. However, Asian seabass still suffer from several major diseases. Among them, viral nervous necrosis (VNN) disease, caused by nervous necrosis virus (NNV), is the most severe one, as it causes more than 90% mortality of Asian seabass larvae and continues to threaten Asian seabass in the juvenile stage^30^. One our previous work used 145 SSR markers to construct a genetic map and several QTL for resistance against VNN were identified^25^. However, these QTL still have large confidence intervals, making it hard to use these markers in the MAS program in Asian seabass. Moreover, dissection of QTL and identification of genes or genetic polymorphisms underlying these QTL are impossible due to the large region in the chromosome.

To fine map these QTL and further identify candidate genes underlying the QTL, we employed GBS and identified 6425 SNPs. We constructed a high-density genetic map consisting of 2852 SNPs, and 148 SSR markers from a previous study^25^. We further conducted QTL mapping analysis and identified four QTL for resistance and survival time, with phenotypic variation explained (PVE) ranging from 7.8 to 11.0%. We further identified a candidate gene, protocadherin alpha-C 2-like (*Pcdhαc2*), underpinning the QTL of *qNNV-Re_20.1* and *qNNV-Su_20.1*. It was significantly associated with the phenotype. Furthermore, its expression levels in the brain, kidney, muscle and skin were significantly up-regulated in the NNV challenged Asian seabass. Our results could facilitate MAS in selective breeding schemes for disease resistance and set up the foundation for further detailed functional analysis of the potential candidate genes for VNN resistance in Asian seabass.

## Results and Discussion

### Discovering and genotyping of genome-wide SNPs

With high efficiency and low cost, NGS technology has revolutionized the way how the polymorphic markers are developed and genotyped^6^. Using GBS, we sequenced four libraries consisting of each 95 of early dead fish and randomly selected surviving fish and two parents. Each of the two categories (dead and surviving) was subdivided into two subgroups. Each subgroup was used to construct a library. These fish with extreme phenotypes could provide strong QTL mapping power^31^. After reads processing, including removing low quality reads, trimming and filtering missing genotype and offspring, a total of 784.68 million high-quality clean reads were obtained. Of these filtered reads, an average of 4.36 million reads were assigned to each offspring, 14.74 million and 19.05 million reads were assigned to sire and dam, respectively. Using the clean reads from parents, a catalogue consisting of 18857 loci was obtained. This catalogue was used as a reference to obtain the SNPs and genotypes of the mapping population. A total of 6425 SNPs was identified in 85 dead and 94 surviving fish with the filtering criteria of <20% missing data across all the samples and >5x coverage for each data point. The sample size of 179 fish for fine mapping QTL in this work is higher than our previous study of 144 individuals, and enabled us to successfully identified a gene, peroxisomal acyl-coenzyme A oxidase 1 (*Acox1*), located in a major QTL for growth in Asian seabass^12^.

### Construction of a high-density linkage map

**Figure 1:**
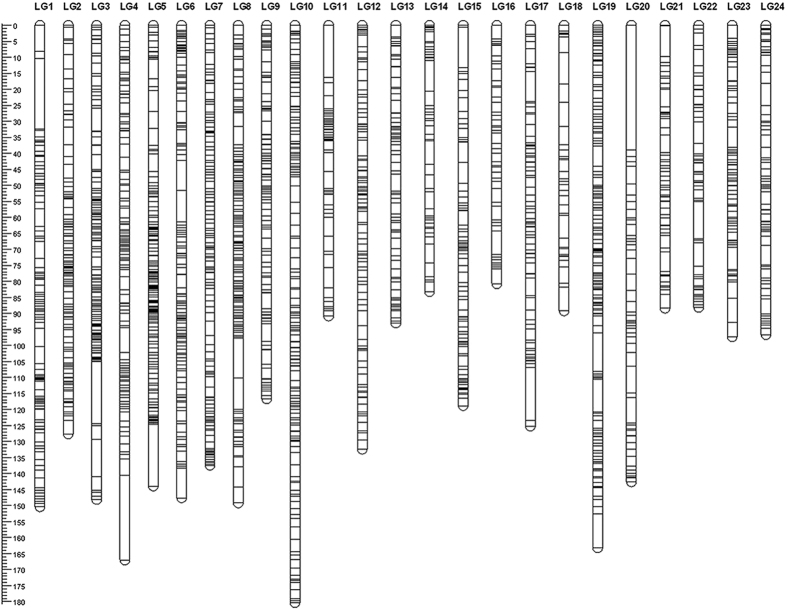
The linkage map of Asian seabass containing 24 linkage groups.

A linkage map is essential for downstream analysis like QTL mapping. In order to construct a linkage map, all the 6425 SNPs discovered from GBS and 155 SSRs from our previous study^25^ were assessed for Mendelian segregation by Joinmap 4.1^32^ before map construction. After removal of distorted markers, 3017 SNPs and 155 SSRs were used to construct a genetic map. Among these, a total of 2852 SNPs, and 148 SSRs, were successfully mapped to the genetic map. This high-density map consisted of 24 LGs (Fig. 1), contained 3000 markers (Supplementary Table S1) and spanned 2957.79 cM with an average marker interval of 1.27 cM (Table 1 and Supplementary Table S1). In this linkage map, a total of 670 markers were observed to be clustered together at 366 positions across the 24 LGs and co-segregated in groups (Table 1). No recombination happened between these markers, thus the marker interval was zero. Although these markers were known to be clustered together in the map positions, their orientations were hard to determine. This is referred as bin signature^33^. Similar findings of bin signature has also been reported in the linkage map of Pacific white shrimp^11^. Several reasons could contribute to this, including the very close physical positions of these markers in the same chromosome resulting in nearly no recombination. Alternatively, these markers could be physically distant from each other but located in the cold spots of recombination^34^. Additionally, a relatively small mapping population (e.g. 179 individuals in this study) could limit the detection of recombination events during meiosis. Thus, increasing the number of individuals could increase the power to detect recombination between markers as well as to determine their orientations in the bin signature. Nevertheless, the quality of this map is much higher than our previous map for QTL mapping for NNV resistance in terms of marker number (3000 vs 145) and density (1.27 vs 6.86 cM)^25^. This demonstrates that GBS has the ability to robustly identify a larger number of high-quality and high-confidence markers than conventional SSR marker discovery, for construction of genetic maps in aquatic species. This further indicates that the present map has much more power for QTL mapping than the previous SSR marker based map^25^, increasing the capability to capture QTL while reducing the possibility of false positive QTL. The higher resolution of the map could also narrow down the QTL confidence interval. Nevertheless, the number of mapped SNPs was slightly less than the 3321 SNPs produced by GBS in a map for QTL mapping for growth in Asian seabass^12^. A possible reason could be due to the differences of cross design. For example, we used a backcross population of 179 individuals while the previous map used an F_2_ population of 144 for the map^12^. It is obvious that backcross could reduce the genetic variance because one fourth of homozygous genotypes were absent in the offspring population compared to the F_2_ population. Therefore, this absence has translated into the reduced number of polymorphic SNPs.

In addition, the total length of the present genetic map (2957.79 cM) was longer and its average marker interval was larger, than those in the previous map of 1577.67 cM and 0.52 cM^12^, respectively. It is known that the genetic map is built on chromosome recombination during meiosis. There is a common difference in recombination frequencies between sexes of fish species, including Asian seabass, in which the length of female LGs was longer than that of male^12^,^18^. It is straight forward that the longer the linkage map is, the less recombination, under a comparable number of genetic markers in the same species. However, the reasons for the huge difference in total length between our two linkage maps in Asian seabass remain unclear. It could be due to the different families used for constructing the genetic maps and further genetic map analysis on more families of Asian seabass could clear this suspicion.

The length of each LG ranged from 80.70 (LG15) to 180.30 (LG8) cM with an average length of 123.24 cM (Table 1). The number of markers in each LG varied from 43 (LG6) to 233 (LG21) with an average number of 125. The marker interval of each LG ranged from 0.53 (LG19) to 4.03 (LG18) cM with an average of 1.27 cM. This small average marker interval could substantially improve the map resolution, which naturally enhances the effectiveness of fine mapping. The largest marker interval gap was 38.95 cM, located in LG 20. Furthermore, marker intervals were not consistent across all the LGs. We also compared this map with the previous one^12^ which was constructed by markers identified by the same method of GBS, and found that there were differences in length, number of markers and average marker interval in each LG. This could be a result of different families, different genomic structure of the chromosomes and/or the specific sequences produced by enzyme digestion. Further studies of mapping those reads to the genome reference of Asian seabass could answer this question.

### Mapping QTL for resistance to VNN disease

**Figure 2:**
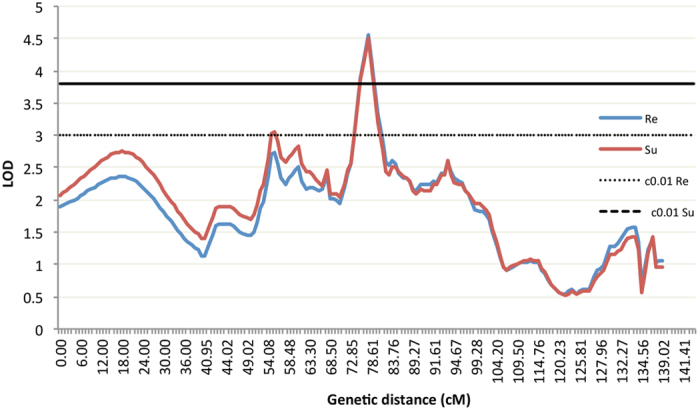
Two significant QTL of *qVNN-Re_20.1* and *qVNN-Su_20.1* detected in LG 20 of Asian seabass, Re for resistance and Su for survival time, c0.01Re and c0.01Su for 0.01 significant level on chromosome-wide.

A high-resolution genetic map could improve the capability to map high-confidence QTL and narrow down the large interval of QTL to a relatively small one, facilitating identification of causative polymorphisms responsible for the QTL. In order to identify QTL related to NNV resistance in Asian seabass, we performed QTL mapping using the high-resolution map and trait values for resistance (Re) and survival time (Su). QTL mapping analysis resulted in four QTL being detected in three LGs (4, 10 and 20) for Re (Table 2), and another four QTL being mapped in four LGs (4, 10, 20 and 23) for Su (Table 3). In contrast, our previous results showed that thirteen QTL in nine LGs and ten QTL in six LGs were identified for Re and Su, respectively^25^. The differences could be caused by the low-density map, which may have resulted in possible false positives in the previous study^35^. This also highlights that a high-density map could yield more creditable QTL. For resistance, *qNNV-Re_20.1* located in LG 20 was detected as significant with a PVE of 11.0% (Fig. 2 and Table 2), the highest among all the detected QTL. It was the same QTL detected in the previous study which also explained the highest proportion of phenotypic variance 4.1%^25^. In this study, this QTL spanned 1.76 cM from 76.85 to 78.61 cM with peak position at 77.61 cM, where the SSR marker LcaTe0441 is located, in LG 20 (Table 2). In contrast, this QTL spanned a region of 3 cM in the previous map^25^, much larger than in the current study. This clearly demonstrates that the high-density map of the current study has dramatically narrowed down the QTL region to a small confidence interval while increasing the PVE explained by the same QTL. In addition, two more suggestive QTL were detected in LG 10 with relatively small confidence interval (Table 2). It again shows that the current map could yield more accurate QTL mapping.

For survival time, two significant and other two suggestive QTL were detected in four LGs. One significant QTL *qNNV-Su_20.1*, with the highest PVE of 10.9%, was detected in LG 20 (Fig. 2 and Table 3). It spanned 3.16 cM from 75.85 to 78.61 cM with SSR marker LcaTe0441 at the peak position of 77.61 cM. This QTL was repeatedly identified as significant with the highest PVE for Re and Su in both current and previous studies^25^. It not only highlights the cross-validation of this QTL by two studies, but also strongly suggests that the same genes or pathways could be responsible for both resistance and survival time. This overlapping by Re and Su was also noticed by several QTL studies for resistance to viruses^36^, bacteria^37^ and parasites^38^ in turbot (*Scophthalmus maximu*). Furthermore, the different QTL mapped for Re and Su could reflect the genes involved different aspects and development stages of disease^39^.

In the present study, we have mapped multiple loci explaining relatively small to moderate proportions of phenotypic variance for VNN disease resistance in Asian seabass. This reflects the polygenetic nature of quantitative traits, which is in line with the classical quantitative genetics theory^31^. Similar results were also reported in other aquatic species for disease resistance, such as QTL in Atlantic salmon^40^, turbot^36^,^37^,^38^, eastern oyster (*Crassostrea virginica*)^41^ for resistance against viruses, bacteria and parasites. However, we also noticed that several studies reported major QTL for resistance to infectious pancreatic necrosis (IPN) virus^42^,^43^,^44^ and salmonid alphavirus^45^, explaining up to 50% phenotypic variation, in Atlantic salmon. Due to their large effect on the trait, these QTL have already been applied in MAS in Atlantic salmon, greatly reducing the economic losses of the salmon industry^46^. The failure of mapping major QTL in our study could be attributed to a single family being used for mapping, which probably does not have major QTL, or due to no major QTL in Asian seabass population. Multi-family screening could increase the possibility to detect major QTL because a large number of individuals with extreme traits could exist in the mapping populations, thus increasing the detection power for major QTL. In addition, mapping QTL in multi-family would allow precise mapping as the LD block is smaller and cross-validation of these QTL to reduce the false positives. This was demonstrated in Atlantic salmon, as most of these studies were performed on multi-family^43^,^44^,^45^,^47^. Therefore, future studies should focus on QTL mapping on multi-family using GBS, which could allow detection of common QTL for resistance to VNN in Asian seabass. It is worth noting that even if the trait values of parents in the present study were unknown, it would still be feasible to use their offspring to conduct QTL mapping. This is because the reassortment and recombination of different alleles in the offspring population could produce a range of phenotypic values^48^.

As the QTL tracked in this study could only explain up to 37.0% and 37.9% of phenotypic variation for resistance and survival time (Tables 2 and 3), respectively, the vast majority of missing PVE was not assigned to any QTL. This could be a result of the stringent threshold set by the current QTL mapping approach which could filter out many QTL with very small effects that might explain the missing PVE. GS could overcome this barrier to capture all the QTL with small, moderate and large effect^4^. GS refers to estimation of genomic breeding values of selected candidate using genome-wide high-density genetic markers, with an assumption that all the causative QTL are in LD with at least one genetic marker^4^. Accurate prediction of the genomic estimated breeding values (GEBV) requires a considerable number of genome-wide markers, preferably SNPs, and genotypes of a large training population^4^. GBS, possessing the capability to produce tens of thousands of cheap SNPs in a large population, in combination with genotype imputation within the linkage block to reduce the genotyping cost, has the great potential to be applied in aquaculture breeding programs^49^,^50^. With the aforementioned merits, GS is becoming a powerful tool in selective breeding in livestock like dairy cattle, sheep, pig and other domesticated animals, greatly improving their genetic gain in a reduced period in the past decade^3^. Therefore, future studies could include GS to accelerate the genetic gain for disease resistance in Asian seabass.

### Identification of a candidate gene underlying the significant QTL of *qNNV-Re_20.1* and *qNNV-Su_20.1*

Fine mapping QTL with a relatively large effect in a narrow region and using the polymorphism in LD with the corresponding QTL are plausible in LD-MAS. However, LD decays over generations at various degrees and thus compromises the effectiveness of MAS^31^. Moreover, little information could be presented for understanding of the mechanism of disease resistance unless the causative polymorphisms underlying gene or regulatory region variance were identified^31^. Therefore, translation of QTL into causative genes containing the QTN could be essential for comprehensive understanding the mechanism of disease resistance. Thus, in order to identify candidate genes in the identified QTL region, we mapped the transcriptome of Asian seabass^51^ to the corresponding genomic DNA of *qNNV-Re_20.1* covering 300 kb using GMAP^52^. The result showed that there were 62 predicted genes in this region (Supplementary Table S1). After careful comparison and consideration of the potential functions of 62 genes, a candidate gene protocadherin alpha-C 2-like (*Pcdhαc2*), was proposed to be the possible causative gene controlling *qNNV-Re_20.1*. However, we can not rule out the possibility of other genes being the candidate gene underlying QTL of *qNNV-Re_20.1* and *qNNV-Su_20.1.* Interestingly, a recent study showed that the causative gene underlying a major QTL for resistance to the IPN virus in Atlantic salmon was the epithelial cadherin (*Ecdh*) gene^46^. *Ecdh* is a calcium-dependent cell-cell adhesion molecule with versatile functions in epithelial cell behavior, tissue formation, cancer suppression, as well as receptor for pathogens^53^. There was a missense mutation in the coding region of *Ecdh*, explaining a majority of phenotypic variation^46^. Further study showed that *Ecdh* bound to IPN virions, facilitating the internalization of the virus in the susceptible Atlantic salmon individuals while preventing the virus internalization in resistant ones^46^. Surprisingly, *Pcdhαc2,* together with *Ecdh*, belongs to the cadherin superfamly^54^.

**Figure 3:**
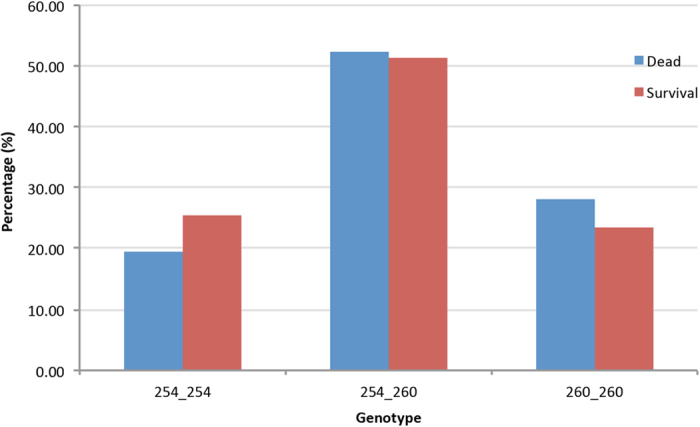
Association between genotypes of *Pcdhαc2* and phenotype in the current mapping population of Asians seabass.

This aroused our speculation that *Pcdhαc2* may play a role during the interaction between NNV and Asian seabass. Therefore, we first examined the cDNA sequence of *Pcdhαc2* (Supplementary Fig. S1) by retrieving it from the Asian seabass transcriptome^51^. This sequence, 7 kb long, contained an ORF of 3 kb long encoding 999 amino acids, and consisting of four exons (Supplementary Figs S1 and S2). Next, we examined the coding sequence (CDS) of *Pcdhαc2* in the two Asian seabass parents, and found no SNPs in any of the four exons, making it impossible to use SNPs to examine the association between this gene and trait. We further examined the genomic sequence of *Pcdhαc2* in the parents and found that there was a six bp insertion-deletion (InDel) and two nucleotides mutations in the 3181 bp of the 2^th^ intron (Supplementary Figs S2 and S3). In order to conduct association study of *Pcdhαc2* to phenotype, a pair of primers was designed for targeting the six bp InDel. In addition, an association mapping population from a mass cross was also developed. This association population consisted of 1127 individuals (476 survival and 651 mortalities) from 43 families of 15 parents. Capillary gel electrophoresis of the fluorescence labeled DNA fragments showed that length of PCR product was 254 and 260 bp. The association study of genotypes with phenotypes by Chi-square test showed that the InDel of *Pcdhαc2* is significantly associated with disease resistance (*p* = 0.0325). The proportions of individuals in mortality and survival groups with genotype 254_254 were 19.51 and 25.42%, respectively. While for genotype 260_260, the proportions for mortality and survival were 28.11 and 23.32%, respectively (Fig. 3). Besides genotypic analysis, the allelic test showed that there was significant difference in the allele frequencies between the survival and mortality groups (*p* = 0.0120). The frequencies of 254 and 260 in the survival group were 51.05% and 48.95%, respectively; those in the mortality group were 45.70% and 54.31%, respectively. These results could indicate that genotype 254 may be the resistant allele, while 260 may be the susceptible allele. It could further indicate that the 6 bp deletion is associated with the increased survival proportion. The possible reason could be that InDel in the intron could influence the mRNA transcription, splicing, stability and degradation, and eventually phenotypic expression^3^. However, it is impossible to identify the exact cause with our current data. Further study is required to understand the function of the 6 bp InDel in *Pcdhαc2.*

**Figure 4:**
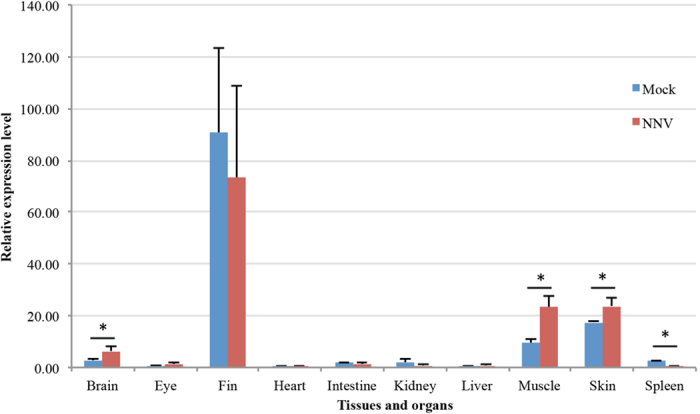
Expression pattern of *Pcdhαc2* gene in ten tissues and organs at 5 dpc in mock and NNV-challenged Asian seabass.

The association between the genotype of *Pcdhαc2* and phenotype aroused our interest in examining the expression level of this gene in the mock and NNV-challenged fish. We conducted qRT-PCR to determine the expression of *Pcdhαc2* in ten tissues and organs: brain, eye, fin, heart, intestine, kidney, liver, muscle, skin and spleen of NNV-challenged and mock Asian seabass at 5 day-post challenge (dpc) (Fig. 4). The result showed that *Pcdhαc2* was significantly induced in the brain (*p* = 0.0189), muscle (*p* = 0.0027) and skin (*p* = 0.0164) after NNV infection, while was suppressed in spleen (*p* = 0.0013). This indirectly indicates that *Pcdhαc2* may play a role in NNV-Asian seabass interaction. Whether the *Pcdhαc2* binds to the virion of NNV, thus playing a similar role to *Ecdh* in Atlantic salmon, or plays a different role, is still unknown. Further studies will focus on elucidating the exact function of *Pcdhαc2* in NNV Asian seabass interaction.

## Conclusion

In the present study, we constructed a high-density linkage map of Asian seabass consisting of 3000 SNPs and microsatellites. Using this map, we fine mapped four moderate QTL for resistance and survival time which could be used in MAS for VNN resistance in the breeding program of Asian seabass. In addition, we characterized a possible candidate gene, *Pcdhαc2*, underlying *qNNV-Re_20.1* and *qNNV-Su_20.1*, which could provide foundation for further analysis of its function in Asian seabass – NNV interaction. Certainly, other predicated genes in this QTL region should not be neglected. Further fine mapping in a large population may lead to identify the causative polymorphism for VNN resistance.

## Methods

### Ethics Statement

All handling of fish followed the instructions set up by the Institutional Animal Care and Use Committee (IACUC) of the Temasek Life Sciences Laboratory (TLL), Singapore, and the project was approved under the title “Breeding of Asian seabass resistance to viral diseases” (approval number TLL (F)-13-003) by TLL’s IACUC.

### Mapping population and DNA isolation

The mapping population used in this study was originally from a population challenged with NNV as described in details in our previous study^25^. Briefly, about 700 fingerlings (37 days post hatching with an average body weight of 1.00 ± 0.20 g) from a backcross were immersed in seawater containing 9 × 10^6^ TCID_50_/ml of NNV for two hours, before being transferred to clean seawater, and designated as challenged group. A similar procedure was applied to the mock group of Asian seabass, but adding an equivalent volume of used L-15 medium instead of NNV-containing cell culture. The two groups were under close monitoring during the whole experimental period. Dead fish were collected two times every day, kept in pure ethanol and stored at −80 °C for subsequent analysis. Massive mortality (more than four mortalities every day) in the challenged group began at 10 days post challenging (dpc) and diminished to the baseline (less than three mortalities for two consecutive days) at 24 dpc. Subsequently, the whole experiment was terminated when the mortality rate reached approximately 50%. There was no mass mortality observed in the mock group. Examination of NNV by PCR method showed that the Asian seabass fingerlings in the challenged group were dead from NNV infection. The first 94 dead and 94 survived fish plus two parents were selected to form a panel for the downstream analysis. The genomic DNA of the panel was isolated using the salt precipitation method described in ref. 55 and stored at −80 °C until library construction.

### Sequencing library preparation and next generation sequencing

The concentrations of genomic DNA were determined by plate reader of Infinite® M1000 PRO (Tecan, Männedorf, Switzerland) using Qubit® dsDNA HS Assay Kit (Life Technologies, Carlsbad, CA, USA) following the manufacturer’s instructions. The sequencing RAD libraries were prepared using double digestion RAD-seq method with some modifications^56^ as described in our previous paper^12^. In brief, 200 ng of each DNA was digested with 20 units of restriction enzyme of PstI-HF and MspI (New England Biolabs, Ipswich, MA, USA) at 37 °C for 2.5 hours. The DNA fragments were examined by electrophoresis on a 2% agarose gel before ligation with barcoded adaptors^56^. The ligation products were pooled, followed by the size selection of 350 to 600 bp using Pippin Prep (Sage Science, Beverly, MA, USA), and then a cleanup using QIAquick PCR Purification Kit (Qiagen, Hilden, Germany). The total fragments were PCR amplified using Phusion® High-Fidelity DNA Polymerase (New England Biolabs, Ipswich, MA, USA), followed by a second clean up using QIAquick PCR Purification Kit (Qiagen, Hilden, Germany). Quantification of the libraries was determined using KAPA Library Quantification Kits (Kapa Biosystems, Wilmington, MA, USA) by qPCR in the MyiQ Thermal Cycler (Bio-Rad Laboratories, Hercules, CA, USA). The libraries were sequence on NextSeq 500 platform (Illumina, San Diego, CA, USA) to generate raw sequencing single-end reads of 151 bp.

### Processing of NGS reads, identifying and genotyping of SNPs

The raw sequencing reads were processed by the program *process_radtags* implemented in Stacks package (version 1.21)^57^ to remove low quality reads and any uncalled base. In order to reduce the sequencing errors at the end of each read, all the clean reads were trimmed to 95 bp, following a final step of de-multiplexing bioinformatically and assigning clean reads to each sample. All the downstream analysis of stack assembly, sequence mapping, SNP calling and genotyping were performed by the Stacks platform^57^ with parameters described in ref. 12. For the two parents, the stacks and catalogue loci were constructed with a minimum of 20 times coverage^57^. For the offspring, a minimum of five times coverage was applied to assemble the stacks. SNP calling and genotyping were conducted by *sstacks* and *genotypes*^57^, respectively. Any SNP with more than 20% missing data in both genotype and individual were removed from further analysis.

### Construction of a linkage map

All the SNP markers and SSR markers from our previous study were submitted to JoinMap 4.1^32^ to construct a genetic map. The Mendelian segregation distortion of each marker was examined using Chi-square test in JoinMap 4.1^32^ and distorted markers were excluded from further analysis. Linkage relations between markers were analyzed in JoinMap 4.1^32^. Marker orders and positions in the genetic map were determined using maximum likelihood in Kosambi’s model with a minimum logarithm of odds (LOD) of three^32^. The genetic map was visualized using Mapchart (Version 2.2)^58^.

### Mapping QTL for resistance to VNN disease

After linkage analysis, identification and mapping of QTL were carried out by MapQTL 6^59^ using a maximum likelihood (ML) through interval mapping (IM). The confidence intervals were estimated by bootstrapping methods to define the smallest chromosome segment with 95% of the most likely QTL position^60^. The association between marker and QTL was determined by the Kruskal-Wallis analysis^59^. To determine the statistical significance of the QTL signal, the significant threshold of LOD was determined through a simulation with a permutation test of 1000 times for each LG and trait under the null hypothesis of no QTL at a given map position^61^. QTL with LOD scores greater than threshold scores at *P* < 0.05 level at chromosome-wide were considered as suggestive, while greater than threshold scores at *P* < 0.05 and *P* < 0.01 at genome-wide were considered as significant and very significant, respectively.

To determine expression levels of candidate genes underlying the identified QTL in Asian seabass by qRT-PCR, three-month old Asian seabass were transferred from the Marine Aquaculture Center (MAC), Agri-food and Veterinary Authority Singapore (AVA) in St. John Island, to Temasek Life Sciences Laboratory. Prior to challenging, fish were acclimatized in two tanks with 25 liters of circulated seawater (30 °C, salinity 30 ppt, pH 7.6) and saturated oxygen for 5 days. In the whole period of experiment, fish were fed with a commercial feeder (Marubeni Corporation, Tokyo, Japan) twice a day, and half of the seawater was replaced with fresh seawater every two days. At the day of challenge, three fish were randomly selected and weighed with an average body weight was 16.36 ± 3.04 g. During the challenge, one group of fish was each intraperitoneally injected with 0.1 ml of NNV stock with a concentration of 3.75 × 10^8^ TCID_50_, and designed as the NNV-challenged group. The mock group was each intraperitoneally injected with an equivalent amount of 0.1 ml of used L-15 medium. All the fish were closely monitored in the whole experimental period. At 5 dpc, three fish from each of the two groups (NNV-challenged and mock) were scarified, before collection of ten tissues and organs: brain, eye, fin, heart, intestine, kidney, liver, muscle, skin and spleen. Total RNA was isolated from these tissues and organs using TRIzol (Life Technologies, Carlsbad, USA) following the manufacturer’s instruction.

### Expression pattern of a candidate gene located in QTL *qNNV-Re_20.1* and *qNNV-Su_20.1* in tissues and organs

Following the identification of QTL for resistance to VNN, the nearest marker to the peak of QTL was determined and the sequence harboring the SNP or SSR was retrieved. The obtained sequence was used as seed to retrieve 300 kb sequences from the Asian seabass genome, followed by blasting the Asian seabass transcriptome against those sequences by GMAP^52^ with default parameters to identify genes in these QTL. Candidate genes were determined with criteria that genes with the highest score and longest matching length were selected.

To determine the expression pattern of the candidate gene after NNV infection, qRT-PCR was conducted on the RNAs extracted from ten organs and tissues at 5 dpc in the mock and NNV challenged groups. Primer pairs were designed by DNASTAR Lasergene 11 (DNASTAR, Madison, USA) based on the ORF of candidate genes. In this study, *Pcdhαc2* was the possible candidate gene for QTL *qVNN-Re_20.1* and *qVNN-Su_20.1* and primer pairs of 5′-GCTTATGCCCTGCGAGCCTTTGA-3′ and 5′-CATTGCCGGGAGGGTTGACTATCAG-3′ were obtained. Before qRT-PCR, total RNA of each sample was treated with DNase I recombinant RNase-free (Roche, Basel, Swissland) following the manufacturer’s instructions, to remove the potential genomic DNA contamination. A total of two μg of total RNA was used to synthesis cDNA by M-MLV Reverse Transcriptase (Promega, Madison, USA) and 0.5 μg of random hexamer primer. qPCR reactions were performed on Applied Biosystems 7900HT Fast Real-Time PCR System (Applied Biosystems, Foster City, USA), using SYBR Green as fluorescent dye. A 10 μl qPCR reaction contained 1.6 water, 3 μl (15 ng) of 10x diluted cDNA, 0.2 μl (2 μM) of each primer and 5 μl of 2x master mix from KAPA SYBR® FAST qPCR Kits (Life Technologies, Carlsbad, USA). The qPCR followed the conditions of 95 °C for 3 min, 40 cycles of 95 °C for 10 s and 58 °C for 30 s. Each reaction had three repeats. ∆∆Ct method^62^ was used to analyze the relative quantifications and fold change of each gene using the elongation factor 1-alpha 1 (*EF1α1*) as a reference gene^20^.

### Association study of *Pcdhαc2* in multiple families

To verify the *Pcdhαc2* as a candidate gene underlying the QTL, we performed an association study in multiple families of Asian seabass. Primer pairs of Forward 5′-CCGTGCCATGCTGTGAGTGC-3′ and Reverse 5′-GATGCCGGAGCTGTGTTCTGTTA-3′ targeting the 6bp InDel were designed. Forward primer was labeled with fluorescence FAM (Sigma-Aldrich, Missouri, USA) at its 5′ end. An association population of 1127 individuals (476 survival and 651 mortalities) was developed from 43 families, which were produced by a mass cross between 15 brooders. The number of families in this population was estimated using molecular parentage assignment with a multiplex PCR set consisting of 10 primer pairs, which was developed by our lab for parentage assignment. The parentage assignment was conducted as described in ref. 63. The challenge experiment and DNA isolation for the association population was followed the same procedure as described in this section of ‘Mapping population and DNA isolation’. The association population was genotyped by PCR as described in ref. 25. Briefly, a 25 μl of PCR reaction included 1 × PCR buffer, 500 μM of each dNTP, 2 μM of each primer, 2.5 units of Taq polymerase and 30 ng of genomic DNA. The thermal cycling conditions were as follows: 94 °C for 3 min, followed by 36 cycles of 94 °C for 30 s, 58 °C for 30 s and 72 °C for 45 s, with a final extension at 72 °C for 10 min. PCR products were detected using capillary electrophoresis by a 3730xl DNA analyzer (Applied Biosystems, California, USA) and allele sizes were determined by comparison with size standard GS-ROX-500 (Applied Biosystems, California, USA) using the software Genemapper (Applied Biosystems, California, USA).

### Data deposition

All the raw reads were submitted to the sequence reads archive (SRA), NCBI database with an accession number of SRP073060.

## Additional Information

**How to cite this article**: Liu, P. *et al.* Fine mapping QTL for resistance to VNN disease using a high-density linkage map in Asian seabass. *Sci. Rep.* **6**, 32122; doi: 10.1038/srep32122 (2016).

## Supplementary Material

Supplementary Information

Supplementary Data 1

## Figures and Tables

**Table 1:** Statistics of 24 linkage groups in the linkage map of Asian seabass.

Linkage group	No. of markers	No. of markers in bin signature	No. of markers for map statistics	Total length (cM)	Average length (cM)
1	130	23	107	163.19	1.53
2	124	5	119	142.59	1.20
3	233	79	154	90.79	0.59
4	131	11	120	150.39	1.25
5	230	66	164	88.12	0.54
6	159	28	131	89.20	0.68
7	173	36	137	93.02	0.68
8	224	82	142	180.30	1.27
9	130	23	107	127.60	1.19
10	177	32	145	125.17	0.86
11	63	11	52	116.67	2.24
12	122	22	100	88.45	0.88
13	93	18	75	143.93	1.92
14	59	40	19	137.38	7.23
15	117	29	88	80.70	0.92
16	72	15	57	97.35	1.71
17	92	16	76	118.76	1.56
18	43	6	37	149.24	4.03
19	214	58	156	83.17	0.53
20	66	14	52	96.65	1.86
21	78	18	60	148.11	2.47
22	65	15	50	166.96	3.34
23	112	33	79	132.43	1.68
24	93	19	74	147.64	2.00
Total	3000	670	2331	2957.79	
Minimum	43	5	37	80.70	0.53
Maximum	233	82	164	180.30	4.03
Average	125	28	97	123.24	1.27

**Table 2:** Identified QTL for resistance (Re) to VNN in Asian seabass.

LG	QTL	Interval (cM)	Sig.	Threshold LOD	Peak LOD	Peak position (cM)	PVE (%)	Nearest marker	Marker position	K*	Sig.
4	*qVNN-Re_4.1*	41.15–42.15	suc	3.2	3.46	41.15	8.5	LcaTe0075	41.15	13.95	*****
10	*qVNN-Re_10.1*	60.77–70.57	suc	3.0	3.74	62.35	9.2	24304	62.35	10.878	*****
10	*qVNN-Re_10.2*	115.34–116.47	suc	3.0	3.38	115.63	8.3	25617	115.63	11.263	*****
20	*qVNN-Re_20.1*	76.85–78.61	sic	3.9	4.55	77.61	11.0	LcaTe0441	77.61	14.132	******

QTL are significant (sic) and suggestive (suc) at chromosome-wide level, Sig. significant level ***** < 0.001, ****** < 0.0005.

**Table 3:** Identified QTL for survival time (Su) to VNN in Asian seabass.

LG	QTL	Interval (cM)	Sig.	Threshold LOD	Peak LOD	Peak position (cM)	PVE (%)	Nearest marker	Marker position	K*	Sig.
4	*qVNN-Su_4.1*	40.1–43.15	suc	3.1	3.75	41.15	9.2	LcaTe0075	41.15	17.501	******
10	*qVNN-Su_10.1*	62.35–64.35	sic	4.0	4.1	62.35	10.0	24304	62.35	11.44	*****
20	*qVNN-Su_20.1*	75.85–78.61	sic	3.8	4.5	77.61	10.9	LcaTe0441	77.61	10.947	*****
23	*qVNN-Su_23.1*	78.11–78.44	suc	3.0	3.17	78.11	7.8	96495	78.11	0.013	—

QTL are significant (sic) and suggestive (suc) at chromosome-wide level, Sig. significant level ***** < 0.001, ****** < 0.0005.
