# Supplementary material for: Fine mapping QTL for resistance to VNN disease using a high-density linkage map in Asian seabass
Source: Sci Rep. 2016 Aug 24;6:32122. doi: 10.1038/srep32122 (PMC4995370; doi:10.1038/srep32122)
Supplement: Supplementary Information [file srep32122-s1.pdf]

Fine mapping QTL for resistance to VNN disease using a high-density linkage map in Asian seabass

Peng Liu[1,2*], Le Wang[2*], Sek-Man Wong[1,2,3], Gen Hua Yue[1,2,4]

[1]Department of Biological Sciences, National University of Singapore, 14 Science Drive, Singapore 117543

[2]Temasek Life Sciences Laboratory, National University of Singapore, 1 Research Link, Singapore 117604

[3]National University of Singapore Suzhou Research Institute, Suzhou, Jiangsu, China 215123

[4]School of Biological Sciences, Nanyang Technological University, 6 Nanyang Drive, Singapore 637551

*These authors contributed equally to this work

Correspondence and requests for materials should be addressed to S.M.W. (dbswsm@nus.edu.sg) and G.H.Y. (genhua@tll.org.sg)

Supplementary Table S1 Mapped genes in QTL *qVNN-Re_20.1* and *qVNN-Su_20.1*

Start position	End position	Gene ID	Gene description
3524	4762	comp100618_c0_seq1	gamma-aminobutyric acid receptor subunit beta-2-like isoform X3
3633	6613	comp102955_c0_seq1	gamma-aminobutyric acid receptor subunit beta-4-like isoform X3
3633	6613	comp112774_c0_seq1	gamma-aminobutyric acid receptor subunit beta-4-like isoform X1
22691	28526	comp104236_c0_seq1	immunoglobulin superfamily member 11-like isoform X2
31336	35156	comp118174_c0_seq1	trimethyllysine dioxygenase, mitochondrial-like
46607	47364	comp118073_c0_seq1	55 kDa erythrocyte membrane protein-like isoform 1
50894	59003	comp111512_c0_seq1	vesicle-associated membrane protein 2-like
56267	56421	comp111899_c0_seq1	vesicle-associated membrane protein 3-like
66680	72641	comp103891_c0_seq1	procollagen C-endopeptidase enhancer 1-like
74272	76706	comp116332_c0_seq1	transmembrane protein 88-like
90220	90671	comp100192_c0_seq1	uncharacterized protein LOC100699848
113447	114797	comp114137_c0_seq1	neuroligin-2-like isoform X1
114438	114803	comp102074_c0_seq1	neuroligin-3-like isoform 1
115861	132524	comp105962_c0_seq1	neuroligin-4, X-linked-like
196101	224163	comp107627_c0_seq1	fibroblast growth factor 11-like
215185	223970	comp108047_c0_seq1	fibroblast growth factor 13-like isoform X2
223787	223946	comp115888_c0_seq1	fibroblast growth factor 12-like isoform X2
241701	244312	comp110927_c0_seq1	claudin-7-A-like
258588	268224	comp114045_c0_seq1	Kv channel-interacting protein 1-like isoform X2
260901	261109	comp100688_c0_seq1	Kv channel-interacting protein 2-like isoform X5
313890	315376	comp108700_c0_seq1	uncharacterized threonine-rich GPI-anchored glycoprotein PJ4664.02-like isoform X2
322438	342766	comp100349_c0_seq1	protocadherin alpha-C2-like isoform X1
326222	333568	comp101016_c0_seq1	protocadherin alpha-C2-like
359615	360003	comp120288_c0_seq1	alpha-N-acetylgalactosaminide alpha-2,6-sialyltransferase 1-like
366462	366679	comp114506_c0_seq1	protocadherin-10-like
685171	687551	comp101542_c0_seq1	roundabout homolog 2-like isoform X3
724161	728058	comp102664_c0_seq1	roundabout homolog 1-like isoform X1
778515	778692	comp106378_c0_seq1	dual specificity protein phosphatase 26-like
812677	819802	comp111020_c0_seq1	protein phosphatase 1D-like isoform X2
812677	819802	comp115610_c0_seq1	protein phosphatase 1D-like isoform X1
840983	842779	comp102668_c0_seq1	T-box transcription factor TBX2b-like
841705	842601	comp121922_c0_seq1	T-box transcription factor TBX2b-like isoform X2
870570	882405	comp114157_c0_seq1	LOW QUALITY PROTEIN acetyl-CoA carboxylase 1
896691	898033	comp111297_c0_seq1	LIM/homeobox protein Lhx1-like
931255	1017849	comp112891_c0_seq1	seizure protein 6 homolog isoform X2
942651	943876	comp103824_c0_seq1	seizure protein 6 homolog isoform X3
1043047	1104197	comp108941_c0_seq1	unconventional myosin-XVIIIa-like isoform X1
1046799	1054297	comp106088_c0_seq1	unconventional myosin-XVIIIa-like isoform X2
1086700	1087892	comp120244_c0_seq1	unconventional myosin-XVIIIa-like isoform X4
1121076	1121732	comp119167_c0_seq1	vascular endothelial zinc finger 1-like

Continued Supplementary Table S1

Start position	End position	Gene ID	Gene description
1121684	1151474	comp106090_c0_seq1	vascular endothelial zinc finger 1-like isoform X3
1129704	1129873	comp104531_c0_seq1	uncharacterized protein LOC100698112
1132670	1155774	comp117524_c0_seq1	kinase suppressor of Ras 1-like isoform X1
1151124	1153978	comp118797_c0_seq1	kinase suppressor of Ras 1-like isoform X4
1168509	1168717	comp100167_c0_seq1	oligodendrocyte-myelin glycoprotein-like isoform X2
1179136	1251492	comp117562_c0_seq1	RNA-binding protein Musashi homolog 2-like isoform X2
1179136	1395096	comp109218_c0_seq1	RNA-binding protein Musashi homolog 1-like isoform X1
1179178	1373848	comp121322_c0_seq1	RNA-binding protein Musashi homolog 1-like isoformX1
1348650	1388120	comp110235_c0_seq1	RNA-binding protein Musashi homolog 2-like
1413099	1420985	comp104342_c0_seq1	nuclear fragile X mental retardation-interacting protein 2-like isoform X1
1416673	1420818	comp102552_c0_seq1	nuclear fragile X mental retardation-interacting protein 2-like isoform X2
1428170	1428365	comp100133_c0_seq1	glycine receptor subunit alpha-1-like
1438969	1440907	comp113275_c0_seq1	microtubule-associated protein futsch-like isoform X2
1445850	1454704	comp105529_c0_seq1	GRB2-associated-binding protein 3-like isoform X1
1457123	1462252	comp104216_c0_seq1	APC membrane recruitment protein 1-like
1469590	1471219	comp113498_c0_seq1	probable ribonuclease ZC3H12B isoform X3
1482722	1494229	comp100770_c0_seq1	ezrin-like
1493123	1493961	comp114850_c0_seq1	moesin-like isoform X3
1531568	1531784	comp108517_c0_seq1	peroxisomal membrane protein 11A-like isoform X2
1560804	1563590	comp101890_c0_seq1	oligophrenin-1-like
1580156	1582678	comp101280_c0_seq1	gap junction beta-1 protein-like isoform X1
1581932	1582600	comp107460_c0_seq1	gap junction beta-1 protein-like isoform X2
1587363	1587666	comp114285_c0_seq1	gap junction alpha-3 protein-like isoform X2
1600115	1600293	comp116137_c0_seq1	TBC1 domain family member 9B-like isoform X1
1600997	1608165	comp116136_c0_seq1	TBC1 domain family member 8B isoform X2
1623120	1627089	comp104106_c0_seq1	neuronal PAS domain-containing protein 2-like isoform X1
1625778	1627050	comp101733_c0_seq1	neuronal PAS domain-containing protein 2-like isoform X2
1676848	1677027	comp108182_c0_seq1	ribosomal RNA processing protein 36 homolog isoform X2
1683207	1683663	comp105941_c0_seq1	uncharacterized protein LOC100690689
1690048	1693564	comp106430_c0_seq1	charged multivesicular body protein 1b-like isoform X2
1696378	1700448	comp105996_c0_seq1	ras-related GTP-binding protein A-like
1703034	1703271	comp114523_c0_seq1	cyclic nucleotide-gated channel rod photoreceptor subunit alpha-like
1703163	1703432	comp121300_c0_seq1	cyclic nucleotide-gated cation channel-like
1710858	1725920	comp117994_c0_seq1	plastin-3
1732103	1741208	comp120629_c0_seq1	transcriptional regulator ATRX-like isoform X1
1732398	1733573	comp110609_c0_seq1	transcriptional regulator ATRX-like
1753579	1753906	comp109943_c0_seq1	coiled-coil domain-containing protein 61-like isoform 2
1758454	1758603	comp119324_c0_seq1	inositol-trisphosphate 3-kinase C-like isoform X2
1769617	1770570	comp111828_c0_seq1	protein phosphatase 1B-like isoform X1
1792648	1797614	comp101920_c0_seq1	poliovirus receptor-related protein 3-like isoform X3
1817525	1822101	comp112361_c0_seq1	protein phosphatase methylesterase 1-like isoform X2

Continued Supplementary Table S1

Start position	End position	Gene ID	Gene description
1818191	1819138	comp110475_c0_seq1	protein phosphatase methylesterase 1-like isoform X1
1826714	1832470	comp115056_c0_seq1	mitochondrial uncoupling protein 2-like isoform X2
1832160	1832326	comp115687_c0_seq1	mitochondrial uncoupling protein 2-like
1838966	1843660	comp100725_c0_seq1	ras-related protein Rab-6A-like isoform X2
1842479	1847306	comp112775_c0_seq1	ras-related protein Rab-6A-like isoform X1
1842981	1847278	comp101509_c0_seq1	ras-related protein Rab-6A-like isoform X3
1852186	1867886	comp113769_c0_seq1	gap junction delta-2 protein-like isoform X1
1867754	1868066	comp102531_c0_seq1	gap junction delta-2 protein-like isoform X2
1897819	1899773	comp116638_c0_seq1	platelet-activating factor acetylhydrolase IB subunit beta-like
1904574	1905828	comp120054_c0_seq1	uncharacterized protein LOC102075665
1907972	1911449	comp115228_c0_seq1	myelin protein zero-like protein 2-like
1922886	1923078	comp121806_c0_seq1	sodium channel subunit beta-4-like
1970154	1974162	comp113144_c0_seq1	Down syndrome cell adhesion molecule-like protein 1-like
1977389	1977667	comp105028_c0_seq1	Down syndrome cell adhesion molecule-like isoform X1
1985050	1999686	comp103373_c0_seq1	rho GTPase-activating protein 35-like

Supplementary Fig. S1 Genomic DNA sequence of *protocadherin alpha-C 2-like*, sequences of yellow highlighting represent UTR, sequences of green highlighting represent exons, sequences with no highlighting represent introns

5′-
GCCTTGACGGATCTCACAGCTCCAGCGGCAGGGAAACACACCCGTCAGCAGCGCGCATTGGAGACAAAAAAGCGCATCACGGACAAAGGAAATATGGGTGCGCTGGAGAGGATTCAC
CTCAGCCGGGACACACAATGGATGAGCCGCTTCTGTTTCGTTTACCGGGAGACCAATTAGCCCACCTTAATGAGGTGCTGGGATGCAAGATACTGCAAGCTAAAAAGGCGGAAGGCTG
CGTTTAATCCGTCTGCGGATCCTATGAGCTTGCAATGGCTGTGGCGGGGATATGCAACTGGCTAGGGAATAATGTGCCTTTTTATTTTGTGATATTTTCCTTATTTTGTGGCCTTTCGTGT
GGACAATTACGATATTCTATTACGGAAGAACTAGAAAATGGGGCACTGGTCGGTGATCTGGCGCATGACTTGGGGCTGGATATTCGAAAGCTCGCCACCCGAAAAATTAAGGTAACCT
CCAACAGCGGTAAACGCTACGTGATTGTCAACCCCAAAAATGGGAAATTACTTGTCAATGAAAGGATCGACAGGGAAGCACTGTGCGATTTATCCAGCACCTGCCTCATTAATCTGGAG
GTGCTGGTCGAAAACCCCACTGAGGTGCACCATGTCGAGGTGGAGATCGTGGATGCCAATGATAATGCGCCGCAGTTCCCCAGAGACGAGTATCAACTGGAAATCACAGAATCTGCTT
TACCGGGGTCTCGTTACCCGATTGAAAACGCTCAGGACCCAGACATAGGGTCCAATTCAGTTCGTATGTATCAGCTCAGCACAAACGACCATTTCGCGCTGGTATCTAACAAACCCTCCC
TAAATACAAAGCACATCGAGCTTGTGCTCAAAAAGCCCCTTGATCGTGAACAGACGCCTTACCACCAATTAATTATAAGTGCTGTGGATGGTGGGACACCAGAGAAAACGGGCACTGCT
AAAATCAACGTCCGGGTCCTGGACTCAAATGACAATGTTCCCTTGTTCGACAGCTCAGTGTACAAGGTGAAGCTGTTGGAAAATTCACCCAAAGACACCCTGGTAATTAAACTGAATGC
AACGGATCTGGATGAGGGCACAAATGGAGAGGTTTATTACTCCTTCAGCAGCTACACTCCAGAGAGAGTGAGGCAGATGTTCAGCATGGACACTAATACAGGAGAAATAAGAGTGAG
GAGCAATGTTGACTATGAAGAGACCAACTCCTATGAGATGTACATCCAGGCGATGGACAAGGGACCTGGGGCCGTGGCAGCGCACTGCAAGGTAGTGGTGGAGGTGGTGGATGTGA
ATGACAACGTCCCTCAGATAGTGCTGTCATCCCTGTCGAGCCCAGTGAGGGAGGACGCCCGCGCAGACACAGTCGTGGCCCTCATTAGCGTTACTGATCGAGACTCTGGCGCGAACAA
GCAGGTGAGCCTAGAGATCCCAGCAGGCCTTCCTTTCAAGATCAAGTCATTCAGAAATTACTACACTCTGGTTACCTCAGCCTTCCTGGACCGTGAGACCACCGATGCCTACAATGTCAC
TTTGAGTGCCACAGACGGAGGCAATCCTCCCCTCTCCTCCCAGAAGACCATACAGGTGGATGTGGCTGATGTTAATGACAACCCACCGCGATTTGAGCAGACTTCATATACAGTCTATGT
GGCTGAGAACAATGCCCCCGGGGCCTCTTTGTGTACGGTGAAAGCTCAAGACGCAGACATCAAAGAGAATGCACGCATCACCTACACAGTGCTCAATGACAACAACCATGGCATCCCT
GTCACCTCGTATGTGTCTGTTAAGGCCGATACAGGGGAGGCTTATGCCCTGCGAGCCTTTGACTATGAGTCACTGAGAGAGTTTCACTTCCAGGTCAAAGCCCAAGATGGGGGCATTCC
TCCTCTAAGCCGCGTCGCCACTGTCTACATCTACATAATGGATCAGAATGACCATGCACCGCTGATAGTCAACCCTCCCGGCAATGGCACACGCTCCTTGGAGACAGTACAAAAGAATGC
AGAGCCTGGTGCCTTGGTGACCAAAGTGGTGGCATATGATGCAGATGCTGGTCCAAATGCCTGGTTGGTGTACGTGCTGGAGACAGCCACTGACCTGGACTTGTTCAAGGTGCACGAA
CACACCGGTGAGATCAGAACCACTCGGAGGATCCTGGAGGACAACTCCACTTCCTTTGCTCTAACCGTCCTAGTGAAGGACCATGGGCAGCCTGCCCTGTCCTCCACCGCCACCATTAAC
GTGGCAGTCATGGAAGTGCCACCCAAAGTGGTCCCTGACCCCAAGAGGATCATGAGACCTCACAGCCCACTGGTCTTCTCCAACGTGACACTGTACTTGATTGTGGCACTGAGCGCCAC
CACCTTTGTTTTCCTGGTCACTGTGGTGGTGCTGGCCATTGTCCGCTGTCACGCCTACTGCACCCAGCCTGGGTCCTGCTCCCCTTGCTGTGTGTCACAGAAGCCCCCTCCAGATGGCGGG
AGCAGCAGTGTAAGTGCAGGTGGGGGAGGCAGCGGTGGAGCCGGGGCACAACCTAATAACAATGTGGTGCTTCGCAGAGACCTTAAAGTGGAGCCTCATTACATCGAAGTGCGTGG
GAATGGCTCCCTAACAAAGACTTACTGCTACAAGACCTGCCTGACAGCCACCTCTGGCAGTGACACTTTCATGTTCTACAACACAGGACGTCCCATCAGTGGCACCTGGGGCAGTGGTG
CTGACCGCTTCTTCACCAGTGGAAGTGGATTTGTACGCAGACTCAGTATGCCTGATGCCTCGCTGCAAATCTGCCCAGAGGTGTGTCATTAATTTTTTATGTTTATCTTTGTGCCATATCTG
GAAGCCCATGAATCAATGTGTTTACATATTAGTCACTTTCTGATGAAATTTTATTTAGTTGCAAACAATATAAAACCCTTTAACTACATACCAAACATGACTTTTCATTAAGGTTATTGCTA
CAGTATGCATTGTTGTCATCATTTTTGTGTGCAGGGTGATATTCCTGCATGTCAAGTTTGATCCCCTCTGGCAATGAATTGAATCCTTGCAGTGCTCATTCTCTTTGTTCAGTTCCCACTTGC
ACCGTGCAGTCACTCGGCCCAGCTCAGCTCAGCGCTCACACTGCATTAGTGTCAGTCCTGATGCCTTTCAAGGTTCCTGTGTTATTTCTGATGATTCAGAGATTTGTGTTTCAGTTTGAGG
TTGTCATCACATCTACACCAAGAACAGGTCATGCTGTCTTCACTGCTTGCTCGCTCTTTGTGCATGTGGGTTTGCCTTGTGTGAGTGTGAGTTAGTGTGAGTGAGTGAGTAAAACTGAGC
GCTGGAGCAGCTGGGGTTAATTGACTTGATCAAGACCACCACAAGACTAGTTGTTGAAGAGGACTTGAGTCAGGCACCCTTGGTTTGCCTGTGTGCCTCTGACTATGGGAGAGATATGC
ATACCTATGACTAGTGCTGATACTGTGAGGAGACTAGATATTGTTTTAGATTTTGTCATTTCACCCTAATTGTTTTCTCCTGATCTAAAAGTATTACACTTGCGTGATACAGTTTTCCTAACT
TCGCATGTGAGTTCTAGATGAGTGAAACCTGCTAGGCAATATCCATACTACTAATGATTTAGTTTCACACACACCAACAACTGATTTAGGTATATTAAAAAATACCTATAGTCATACCTAA
AATACTGTTAATATTGAGGTAGAAATTAAATAATATGACTGTAGTTGGCATGATTACTATTGAAAAGTCTGATACCTGCTTTTCAAGCCATACATGCATCACTTGTGCAGTCCTTAGATAT
TGAAATCCTCATGTCTGTTAGTTTTACCTTTAAGGAGTCTGGGTTTCACTCTAGCAAAGGTATATTCTGCTTTTATGCCTGAGAGAAAGGAGAGATTGACAGTTCAAATATTGGAATAATA
TAGTTTTTATTGCCAGATTATTGCAGCAAGGGCTCCCTAAGGTTAGGAATTTGCCTTTGGATGCATCGTCTTAATTATCAGGTACCAGTCTCTGGTACATTAGTAATACAATGAGAAAATC
TCTTACTGAGACAAGTGTAGCAGTGTAAATTAAATTAACCTCCAGCCTGTGTTCCTCAGCCCTGCAAATTAGATTTACTCAAAGAGGCTGGGTCTACCAGCAAAAAATACTCCACTCTGA
ATTGAACTGAGGAACATGATGTGCCATCTTTATCTGGGACATTTGCCACTGCCTGAGCAGGTTTGTGTCTGAAACACAGCTTTTCTTATTCGGTGTTCTTGAGATCTATGGCTGCAACCCA
CATGATGCTGACAGATTTTAGCATTAGCTTTAATACAAATGTAATAAAAATGTCTTTTCATGTAGTGCATGTGCACAGTTCTCATGGGGGAAAAAAATTATGAAAGGGTCAGGAATAATT
AATGGCCATGAAAGATTGGCTCATTAAAAATATGAACTTTTCCCTCCATTAGGTTCATATGGTTCTTTGAACTTAAATGCATTATCATTAGCAGTCTGGCCATTATCAAAGGTAAATGAGT
GGGGGTTCAGTCCAGTCAAAAGTCATTAAACTAAAATAGGGTAATTTAAAATTCATGTAAAATATTTAGCCAAAAAAATCTTTGTCTAGATAGTGTATTTTAATTTAGCCAATATTCTATA
CTTTCTGTGCTCTTAGAAACATACAAATATCTAACTGTTTATAAGGTTTTTAATGGGAACATATTCCTTTAAAAAATGTGTCTATAGAGTAGTTTCAATGTAATGATGGCAAGGTTTTGGT
GAGAGACAGCCATCAGAATCAATAAATCTCCAAGGCAAGATATATGGAAAAATATTTTATAAAAGATATTTGGATATAATTATAACTATTCAGTGGGCTAATATAGGCCGTGTGTATAG
GATGTGGTTGTCCTAGCGATGATAGCTCTTGCTTACTGGTTTTTAAAGGCATACATTTACCAGAGAGTGGTTAGGAGTTTCAAGCACTCAGCCAAGGTTCATCAGGAGCGCACAGATTG
ACTCCAGTTACAAAAACATATACATCAGTCTGTACAAACACCCCAGCAGGCAGGGAAAGGGCATAACTCTCCCAGCTCTCGCATTTATAGAGCCATAGAGTGAGACTGTCCAAAAACTC
ATCAATATGGCTTTTCAGTCACTGCCCTTTTTAAAGTCCTTGCATGGATGTCAATACGTATGTGTGTGTGTGTATGTGAGTGTGCTTGCAAAGGGGGACACGACTGCAGTGTTCCTACCA
CGTCCAGAGCTGGGATGTGTATCATGATTAATTGATGAGACCATGGCTCAGCCTGGCTCTGCGCTGTATGAGATCAAACTAAACTCACCCTCTT[CCTTCTCTCTCTCTCTCTGTCTCTCCA
TCTTTCTCTTCTCTCTCTACTCTTTTTTCCCCTCTCCTCTCTCTCTCTCTACCTCTTG]CTCGCTCAGCCAAAAGCTCCGAATGCTGACTGGCGATATTCTGCATCTTTGAGGGCAGGGGTGC
AGAGGTAAGCACTTTCCATCTTTAACTCTTTTGCAACTTTTGATCATCTAGAAAAAGTTACACCTATTCATTCTCCTCATATGGTCTAGTGTCTGTGCTACCACTACAGTGACCCTTCATCTT
GCTCTGCAATGTATGGTGATAAATTAGACTTTCTACTGTTACCTGCTCCTCAAAATGACCTCTACTGAAAGGGGAAAAAAACATTGCAGCATAACACTGCAGCTAAGTATCTGTCATTATC
AGAAAACGGGGCTAGTCAACCCTAAGCATCTGTTTGAACCTCAACCCCATCCTCCTCCTTTGGGAAAATCCATGTGCAGCTCCCATCAGTGGATGCAAAGCACTCCTCCTCTGCAGTGTA
ACACTATATAGGCCATCTATATTCTCAGCGTTACTTCCCCCAGTTCCAGTCTTTCAGCATGAAGCATGAAGGACAAATGAGCCGACTATGACCTTTCTCAGATTATCAGACGCACACATCA
TCATCATCAGTGTCTGCACTGCCTGTGTGCAGTTCTGTGTATTTATATATTTTTGTCTAGAGATTAGGGGAGAAATTGTGAAGTCAGACAGTTTAGACTCTGGTCATACTTGTTAATTCAT
AGGACATACTTGCATGCACTCAGTTTTTCCAGCTATGGCTTCATAAGATCTTAAAATCCCATTTACAGATAGGTCTAAAAATACATAAAACATATATACTAAATATTGCACTGAAACTGAA
AGACTTAATTAAGCTTTTAGAACCATGTGTAAATGGTTTCTACACATGATGTAGAACTTCACCTAAAACTGTCAACTCCCAGCAATAATAATGAGCAGGTTCTTGAAATTTGGGTTGCAAG
CTTATGTGGGGTATTATTTCAAATATAGAAAACTGTGTTTTCCATTTGCTTAGAATATAAGTGTTCCTAATTATGTGAGTAAGTAAAGCCAACTACAAATCACAGAATACTGAAACGAAAA
AAAAAGATTTAACAACAATTGGAACAGAATTGTGTTAGTTTTTTTTTTTTCATCTGTAATGAAGTGTTTTGCTAATTTGATAGGACGACAGCTGGAACGTCAGGGACTAATGAGTTTTTCC

TCCACGTCTATCCCTTGTACCGAATTTTCATAATTTCATGCTAACAATGCTTACTGCTCGGTCGTGCAGTCTTCTGCTCCGTCTAAGTTTGATTTAATTGGATATTAGTGTTAATTTCTATGG
TGCGTAGGGTATCATAATGTGCATCATGTCATCAAGCCACTGTAACATGACATGGCTTAACATAAATCATCACTGCAATTTGCCGCTGCCATCTCTAAAATGGCAGTGCCTGTAACACAC
ACAGACACAGAGACGGCTCACATTAACACTTAACCCTAACACACGCACGCTGTAATGTCTCACACTACACGCATCTTGTAGCACCAACATTACTGACCCACATTCCTGGTGTAGTCAGTCA
CTGTAGAGAACACCAGTCCTATCTGTGTCATATTCTCTCACATTAGAACTGCACCTCTGCTTATGTAAAAGCTTGAAACTCACACTGCTCAGGATATACATGGCCATTTTATATTCATGACA
AATCCCTATTCAACATAACAAGTGAGTTGCAGTTTGAGATAGGTACTCGGTGTGCTGCATTGCAGATGCCCCAGTCAGTAGGTCCTGCTATACAGTAAACTCTATATTCATTATGATTGTA
TGAGGGGGGAAAAATCACAGCCTATGTTGGAAACTGCATGCAAGTACTTGGCTTTGTATGTCCATTACAGCCTTACAACAAGGCAACAAGATAACACCCTTAGTAAATGGACATTAACT
ACTCAATAACCAACAGCTTGTCATTTTTTTGCACTGTATGGATTGAACAAACAGTCTGAGAGGTGCTGGTAATTGGATTTTGTGTGTCACATTGGACACAACCAGGCTAGCTGTTTCTGAT
TCCAGTCTTTATGCTTGGCTAAACTAACCAGCTCGCATTATGTGAGTAGACACAAGAGTGGTATCAGTCCTCTCATCTAACTCTAGTTAAGAAAGGGACTGACTGTATTTAGTGTCAAATT
ATTCCTCCTTTTCAACAACCAAAAAAAACCTGTCAACATACAGACCTTTGCACTTTTTTATACAAAAAGTGTAAAAATATAAAGTTTATGATTTTAGGTGGGGGTTAAGTGTGTCTGCTCA
CGAGATGACAAGGCACCAAGAAGTTAATGATTTTGTTATCTTGGGACAGAGCCAGGCCAGCTGTTTCTTTATGCTAAGCTAAGCTAACTGCTCACTGTCACTTTTACAGACAGTTTCAAG
AAATTGAAGTTACAAAATACTTACTGCAGAACACTTTGTTTATTCATTACACTACTAAGAATATTATGCACTGCCAGGGATGTATAAATGACAATTTTCATTTTCATATGAAATGAAAATA
AACTTCTTCAATTCAAGCATGCAAGTAAGTGTCATCACATTGTGAAAGTGAACATTAACAGTGAAAGCAGGAACTGTTTCTGCATTGCCTTCTCTCAGAGTACTGTCTGCCCACCAGCTGT
ATTGAATGAAAAAAGTAGAGCTCAGAAAAGTACACCCTCTGGGTTTTCACATTGGTGAAAAGAAGGAAAAAAAAAAAAACAAACAAACAACAAAAAAAAAAACATCTGAAACTTCAAGA
CAAGAACTCCACTTCGGTTCCATGGCAAGTATCTATGTTTAGACTGTAGTGTGAATTCTTTTTAACATTGTGTCCTCATTTTTATACATGATTATCATATAATAAAATATATGCAGCAAGAT
CAGCATGCAATTAACAACATTAAATATTTATGTATCCCAATATGAGATGAACATTTAGGAGGTATAATAACACATATGCACTGTATATATGTTGATTGCCATTTAGATAAATACTGGAGTT
AAAACCTTACTTGGTAATTCAGAAAACAGAGCATTGTCTTTACATATGTGGTTTTAACCATTAGGGTGGATACCTGTTCTGTACCGTGCCATGCTGTGAGTGCCATGGGTACGACAATGA
CCACTTTTCTATATGTAACTATGGCACAGAAACAGAACCTTCACTTCGTACCTGCATTACATGCTTTTTCCTTTTCCAATCTGCAATACTCAAACCCAATCCAGTTACCAAAAGAATGAAAG
GTCCAAATTTACTGTAGAAATTGACATAAATCAGTTGGTCACTGAAAACTTTCCCTGTGAAGCAGCTGCAGACTGCATTAACAGAACACAGCTCCGGCATCACAACAAAGGTTCATATTT
GTTGTATGAAGGACAAAGCACAGGGGTGAATAATAAAATGAATGATGGCTGAAGTTAATTCAGCAGCTCCAGTTTGGGATGTTTTTTTTTTTTTTTTAAATAAGACAAGTGGGAATCAG
CTGCCAGGGGCAACGTGTCCACACGAAACAAGTGTGACAGTAGTGCAAAACTGGTCGCCCCTGGCAACTAGGCTGTTGAGGCTTGAGGCAGTGACTTAATAGTGCTTGAAGATTTGAC
ACTGTAAGCAATTTTTTGTATTAGATTACCATTTCAGTTGAGGTTTAAAAATACCTAGACTATCAAGGAAAACAAACTTCCATAAGAAGGAATAATTATTAGCTAGAGGTCAGGTCACTG
AATAATAAGTAAATATTTGTTTTTATGTATCTCCTGCCTTTGCTGAATGTGTCCTTTCTTTCAGTTCGGTCCACATGGAGGAGTCGTCTGTGATGCAGGGAGCCCAGGGGATGCTTGTCCA
GAACTGGCCCACTGTTTCTAGTGCTGCAGGTGAGTGGATGAGTAGAAGTCAGAATGTAGGGCAAAAAAGCTTCTTTGATTCTTTGGGGCAATGGTGACTAAAAATATGTAAATTAAAG
GTAACTGATTTTGTCAAGATCACCTGCAAACATTTAAACAGTAGAAAATTTGTTTTTCAAAATATATCAAAAGTATAGAATATTTACTAATATTTAATATCAGGTGTACTAAAATATCTTTA
TGTAATATGCCCTAAAAGCTGGCTGTTAAAAAGCTTGTTAAAGCTTGTATCTCTGTTGCTTTTTATTAGTATATCAATCACACTGTATATTCTTGTACATGATGCAGTTTAGTAAATGACAA
AAAAAGAAAACACGACAGGACAAATATTAAGGTCACTGGATACATGTACTCAGCAACATTAGGCATTTTAGGACCAAACAGTTCTGGGGACGCCCTGAGAGGAACTGCCTGCTGGAGA
GCTCCCCCAAGAGCTACACACCTTAAAGGATTTTTTTTCTGTTCGCATTTGCACCGCCAATAGAAACCCTAAAGTAAGCTGGCCAGCGGTTGGTATAGCAATGAATTTACTCCTCCTGCAT
ATACACTCAAACTCTGGACTTCACTGTTAGTCCATCTGTCTACATCTTGTTCCATTTTGTACAAACTGAAACAAGCGGTTGTTTACACAGCTAACATTTTATATGGGTTTTTTGAAAATGGC
GGTTACTGGTGCTGCACTAGCTCATGTTTGCCCAATCAGCATACATTTAAGTTTGGAGCAATCACTGTATACTTGTATCACTCAGTTCCTCTTGTGCTAGAGACTACCTACTCTGAGGTAG
GGGCTAAAAAGTCCCTCAAACTATGATCATTTCTGATGCTTACAGTACAGACACAACAAAAAGAGGAGGGTCTTCCAATTGTTCTTTGAACTATGAAAAAGTGCCTCCAGTTTGAAAATG
TCTACTGTTACATGATGAAGGCGGGATGACATTTCTAAGTAAAAATTTGAGAAACCGTTGCTATTGGGCTAAATGCAGTGCTGCTTTACAGTAGGCATTCTTCCAAATATAGTACTGAAT
AAAGTTTGGAGTAAAATTCTCACTCACCCCCACCTGGGGTTCAGTCCTCTGTACTGGTATTGGGGGCAGCTGGTACCTTGGTTGTTTGTGCTAAAAAACATAATTAATGGGTGGGTAGA
GCCGGCAGCTGGTTAGCCTAGCTTAAACGCGGCACCCGTCTAATACAATGATGACAGTGATTACACTGAATTCATTTCTGTTTTTACTTTGGTTCAAACAATATGGTAAAACTGGGGATTT
GACAGAATTGAATATCTTCATGATCTTGAATTCCAGTGTGATATTCATACAGAGCTGAGCTGAGATGCCAAAAGCCTCAGGCCCTTGTGCAGAACCACAGATAAACAACTGCATCCTCCC
ATAAAAGCAGCTAGCAGCATGGAGAGCCACAGACAGACAGAAAAAAAAGGCAACTGTTTCTTAATAAGAAATGAGTTGTCCTGAGTGGGTGGCAACACTGAAAATGTGTAGCAGCAGA
ACGTAAGTGTAAATAGAGCTGGCCTTGAAGATGACCTGTGCATCATCATTCACAGAAACACTGGCTCAAAGGTAATGACTAAGAAAATTTGGGAGTACTACATGCATTGTTTGATTGGA
AGACTTTAGTAACTAGGATTTTGTGTTGGTACATTGTGATTCCCTCCTTTCCTTAGCCTAGCTACCATTCATTGATTTTCTGTCTAGCTGCAGGAATTAGAACATACATAGATACTTGAAGT
GTAGCATCACAGTCAATCACTCGACAAGTCAGTGACAGAATATGTCTTACTGTCTTTCAGTGAAAATATATATGGGAATGTTTTTCTCTCTGTGGAAAAGAGAGCTGGGGGATTGTTCAA
GAAACACACTGTTCCAGTTCCCAGTTTCTGTGTCAGTTTGCTGCAAAGAAATGTGCTTTTTGGGGGGGAAATCTGCCTCTCTGGTCATGTTAAGACTCAAATATTGTCTTCGAGAACAGC
ATACTGAAGATGCATCAGGGGAGCATTGTTGCATTAGGAAATAATTTGTTAGGAGTGGTTGAGTATTAAGGCATAGACATGCACTGAAATGCAGGTTGATCCTCTTTAGTATGCATCAT
GAGGTCTTTTCTGTGACACATTTTCATGTCTGCAGTGAATTGTTATCATGATGAAGTTGTCTGATCTGCTGTGATGCATCTAAATGCGGCACCCTCAAAACTGTGTTTATGTAAGTGACCC
ATGGTGCAGATGAGGAGACATTTGTCTAATGCCAGAGGGGGGAGAGGCTGTTATCAGTAATAAAAGAGTGCCA[TGATGAAAAAAAAAAGAGAGAGAGAGATCCAGTAACAT]TAAAA
GCAATTTTGGAAGGGAAGAGCAGAGGAAATGTGCAAGGGAATGGAGGGAAAAAAGCAAGGTGTGGGGGGATGGAGAAAGAGAAAGGTTAAGAGGATTGGCAGGTTCCTGCAGTA
GTGTTGTTGCTAGGGAACGCCATGTGACCTGTTGCCTGGCAACACTGTCTAACTGAGAGATTGCTGCAGCAGCCGCACCCTCCATCAGCAGGGAGTGGGCGAAGGCAGTTTCCATGGC
AACCGGTCATATCTCTGAATGGAAGATTTAGAGACGGAAAAAAGGAAGGAGTAGGGAGCAGAAAAGGGAAGGGGACTCGGCAAGGGCCGGTATTAAGAAGAAAAAGAGGGCACGC
AGTCATTTAGAGGAAATACTGTTAAAAAGACTGGTTTATAGGTCGATGTTTCTCATGCAGAATACATGTGATGCACTGCATACATTTGCACAGGCACTACACGACTGTCAGATGTGTGCA
TGTGCTACAAACCACCAGAGGGATTCATTTTAGATGGATCCGTATCTTGTACTAGACAGAAAATAACGAAGCCTATTTTAAAATGTAGGACACCAGGTTACACGAAATGAGGCTTTAAA
CACCCACATATCTACATCAGGTGTCATGGCAACCTCCTCACAAGGAGCTATACTTTCCTGCTTACCAGCAGTTACACCATAATCCGTTTCAGACACGCATTTGCTGTGAAATACTGGCTAA
TGTGAAGTGATGGCAGAGTTACAAATAATCCTCCATGTCAGAGAGGAAGCAAAGCTCGTCGTAGTTGTACCAACTCCTCCAAGCTGTGACATGACCTGCTCGTTATGAATTAATTTTTCA
CTTGCATGTACACATACACACTCAGACTAATACCTGGGCCAGGATTCTAATTAACAATATTAGGGGTTTGGATTTGTCACAGAAAGGGCAGAAAAGCCTGGGATGTCCCAACACAACAA
GACAATATTCACTTGATTAGACACAACCATATGACAGCTCAAGTGTACTTAAGACAAAAAATGTACTGCTGTGATAGTTTACAATTCTCTTCACTGCAGCAACACAATAATTATAGGGCAT
TTTACAGCATTACTACCAGTCCAGTAATCTTACACACAGTTCCCACTCTCTTAGAAAGCTTTGGTAAAGAATTTATATTAATGTAAGGAACAATTTTAAATAGAATGTTTAACCATATCTTA
GGTTATGTTTACTCTACCACAATAAGTTGTCAAATGCTCAATTAAGCAGGTCATAGCCCACCTGTAATGGTTTGGCCAACACCATTAGATGACCCAATAACCATAGTTTAAGACGGTATGT
TATTATAATGATCAAATCAGGGTAATTACTGGCCAAATTTCTATAAAATCTATAAAATAAGTTAATATTTGTTTGTTACAGAGAATGAATCCAACCGTTTAAAAATTTAACACAAAATATTC
CATAAACTAACAGGCAGAGTGACTGAGCATCAGATCACCAGCAGCATCAGGTAACAGGAAATGATATGTCAGTTATATAAAATGGAGGTGCATTGTTTACAGCAGTGCAATGCAATGC
ATTACTGGCTCAATAAGCTCATCCACATTCTGCTTCATAGTGTTCTGCACATCTCCTTTAACTCACCGGCTCTCTCTTCAGTTTATTTTAAGCGTTTGTCCTCCTCCACATGAGAAGCAATCT
CCCTTGCCTAACCATTGATAGCAATTGAAAGGGACTTATTTCAAATACTGGAACCTCTGCCTGAGTTTAATACCCAGATTAAATACAGACAGACCATACCATCAAATGTCATAATCCTCAG
AAACAGACTACAGATTCAGATTATGCCACTAACTCCACTAATGGTAATATATGGTAATAGTATCTGATACAAGTAATCGCTGAGCAAGCAAAATTACATAAGTCAATTAGCTGTCAATTG
GCTGTCTTTTTTTCTTTTCAAAGAATGTATGTAAAAAAAAAAGAGACAAACTTTGGATTTTCCTTAGAAGCAATGACCTGAGTTCTAGCATGTTGATTTTAGTCTTATCCTGGTCCTTCATA
TGCTGATTTTTTAATGCAAGTTAAAAATGAATAGTTCTATTCTTGCAAGTACAAAACAAATTATTTATGTGTCTTTGTTCTCATGGTCCTGTCACAGTAGTATGTTGCTGCTATGTTACTATG
CCACAATATTGGATCGTACCTCTCCTGCTATATTGAAAACATAAATCCTTTACTGCGTCATATTTACATGAGTTAATACTGTATATGATCAATTTGTAGTGAGTCACGGCTTAATAATTTAT
AACAGTGCTTAAAGGTGTCCAGTGCTCGTTGTGGTCAATATACTGTATGGCTGCAATAATGCAGGGCTATCTATTGACAAGTGTGGTAGTGTCCATTACATCAACTCTTATTACAGCCCA
TACATGTAATGTATTCTCATACTCGATGAACCCACTGTGGGGACTCAGTGGGACACTATGTGTATGGAGAGTGTGTATTTTCAAATTAGTGTACTTCCCTATGGGACAGTGGTTGAAACC
ATTTCAGCACTCCAGCAATGTCATAAATAGCCTATAAAGAAAAATCAAACAGAATCTTCATGCAAGGGCCTGTGTTTGATCTAAATAAAATCCAGACTGAAAGTAGGTCAGTGCAGAGG
GAACTGGTGTGGGCCTGAGAGCACCGTGTCTGCAAAACCCGCCCACCCCCA[TCTTTCTTAGTATCTCTCTCTCTCTCTCTCCCTCTCTCGCTCGCTTTCTTTCTGTGTGTGTGTGTGTGTTT
CTCTCTC]ACTCTTCATCTCATCTAACATCCTCTTTAACCTTTTGACCTTAACAGCTGTCTTATTTTCACTTTACATCATATTTTTTCTATATATAGATCCTATCCTCTATCTTATCCTGTTTGTTT

CTCTGATTTGCTATCTAGGCTCCTTTCTCTGTCACATCCATGCTTGAGCACTAAAGGTTGAATCCCTGTCTCTGGCTTCCTGTGAGGTAGTAGGATTATACTGCTGCCAACATCCAAATACA
AATACAAATCCCATTGCCAAGTTAAGCATTCCCGTAGTTTAAAGGTTAGTCCCCTCCAGTGTTCCCTCCTGTGGTCTCGGCTGAATTGCTGTCCCAAAAATATCCGCTCCCTGTGGCTATTG
CTCCAATGTCAGACTATTTTTCTGCTCGTTGTTGCTTGATTTTGATTATGTGTCCAGTCTTGTATCACCAAATGGCATATGAATAACACGCCCATTTCTTGAAGTCATTTCACATGTTATCAC
AATGCAGTTTTTGCTTTTCGCGTGCCATTTTCTCTACTGAGTAATCCTAACCAAAACAGCATGAAATGACACACAGGATCAAACAGCGTTAATATGTGAAAAGCTTGAAATGTATAGACA
TGACATACAGGAAAGAAAGACAAAAGAATATAGTCTACAGAAGGAACAAATAGGGCAGCTATTGCAAAATGATGCCAAAGCTATAAGGGGATGTGCAAGGGCTGGAGAAAAAAAAA
ATCTGATTAATCACGATTTTTCTTCAAATGATCCAGTACCAATTCTTAAAGTCTTGAGATCGATCTTTGGATTTGTGCTTTTTATCAATAAACATGTATATCTATGTTAAATATTATGGGTAT
GCAGTAGCTACTTGTTGTAGTTAGGCCTGCATCATGTGTAAAGTGCCTGCTTTAGGGAAATCAAGTTAACCTGCACAAAAAGGTTATTTTAAGCCAAAAAGTAACATTAGCCACCTTGGG
TCCTGACAGTCTCATAAACTGTAGCATGAATAGTTACAGTGTGCGAACCTGCTGCAATTTCGCAGGACTGACTATGATGCCGTGGAAGTCAAGGGAACAGTGTGTGGATTCTACTTTATT
CAGTCTGTCCCCACCGGTTAGCTAACACTAGCTAACAGCTGTTTGCAGATATCAGGCTGATGTCTTGTCAACACAAATAGTGCAGATGAATTAGTGAACAGACTTTCAGTTTCTGCTCTGT
CCGTGATCAAAGTGATAATTGAAGTAGATGTCTGTACTTAGTTTATCTGCTACTGACAGTCCATGTATAGCTCTATGGGATAGCATATTTATTTATCTTAAAAATATATAATGATAATTCCC
TTGTAAGTGGAGTAGTTGTTTCTTTATTATTCTTTCTCAACTGTTTTGCACATGTAGACCTAAAAATATGTGTCAGATCAGATCTTGCGGACAGGCAGTCTGCATGGTTATTTTTAAGTGAT
CAGATTGGATTTGTGTGTCCACACATCACCAACCTATTCTGCATGGGTTGCTGTAGGAACGATACAGGTGCCAGTAACTAAACAGCCACCATCACTCTGGATTTGATGATGTTGTGTGTT
GTGTTGTGAGTGATACAAAGACAAGTTCAATTTGAGCAGCAAGATCATTCAAACTGAGACACATCTGTAGAAATCCAACTTGAATCGCATTCCAAGCCACCTACCAATCTTCATGCTAAG
CTAAGCTAGACAAGGGTCATATTTTACTATAGACTTGAGATTTCCCCATCCCACAGAAAATTGAAATGTCAAACTTTTAAGGGAGCTATGTGGTTATAATGATAATTGAAGTAATCTAAG
GATTAGCCAGGTTTGACATGATGTGTTTTCATTTTTTTCCTTCTTACCTGTGTAGATGGAGAAGGTGGTGGAGAGCTTTCACCCCCAGTAGGCGCTGGAGTCAACAGCAACAGCTGGCAC
TTCAGATATGGTGCGGGACAAGGCTATGGCCCTCCTCAGCCCCTGAAGCCTGGAGAGATTCCTCCTGAAGCCTTCATCATCCCTGGATCCCCAGCCATCATTTCTATCCGCCATGACCCA
GGCCCTGTGGATGACAAAGGTGACTTCATAAGCTTCGGCAAAAAGGAGGAGGCCAAGAAGAAGAAAAAGAAGAAGAAGGACAAGAAGGACAAGAAGGAGAAAGGAAAGGATGAG
GGCGGTGAAGAATAGAGGAGAAGGGGTGACTTGAAAACAGAGCTACCAACAAACTATCATCCTAATGTCAGAGCCAGAGAAAAAAAGCTAAATGCATATTGTAGAACCGAGGGAGCT
TCCTTGCACCATATTTGCATAGACACTGTACAGTGGCCAAACCCTCATTAGACCAGGCCTGGCTCCACACATCATGCACACTCGGTAAAACAAATGTCTCCCTCTCTTTCTTGACTCATTTG
GCCCATAACTTTGGCCAAGGTTTACGGCAAACAAATGCTCTTAGTAGCTAGACTTCATCTAAAGGCTGGACCAAATTAAAACAGAAAGTAATTTATTGGATTAGATTAGCCTTTCTGTGA
TTTGCTGCCCCCACCCCCCACCACCACTTTGTATGACGAAGAACAAAAACACCTGAAAAGGTGTACTATTTGTATCTGACCATTGTCAGCTCAGGCTGCCCATGTTGGCATCTCTGTCTTT
ATGCAGCCCCATTCCTTTTTGACTCACCTAGTTGGAGGTGAAGTTGAAGCCAAACTAAAAAAAAAAAAAAAATGAAACCGAGGCTTTTGTCTTCAGTATTATGGAGGCCTTGTAGAAATTC
AGAAAATGTGGTCTTTTATAAAAAATAAATAAATAATATAAAATGAAAAACTGACAACATATAGTATATGTACCATGAGATTAGCACCTTTACACAAATGAGGTGGCTGAGATAAACAG
GACCACACTCGAATAAAGAGCTCTGTTTCAGTGTAACATAGCACATCGCAGAGGCTGAGGACCTGGACTGGGTTGCACCAACTATGCGCAAGTTTGTTCCCAGGTTATGGTATAAAGTA
GTGGCTTCATAACTGCTAGTTCATTTGCAATTAGCAAGTTACTAAATTCAACTGTATAATGGGTACAAAGGGGGGAATTCTATGGCTATAGCTAGTTATAACATGAGAGATTTGAGTAGC
CAGTCAGTAAGAGTACTACCTTGCTGATGGCCATGAATCTCTTTGATTTGTAGTTGACTAGATTGGCATCAAAATTATGATTGGTAAATAGAATCCATATTTGACTCTTAAAGGACAATAT
AGTTTTTGCATGTTTTAGTCCATTTGCTACAAATCATGTCATAACTAACATCACTTCTAACCATTTTCCAAAGCACACCATTGTGTTTTGGGAATTTCAGTGTGTTTCTCCAGCCTTCCAGGC
AGCCATTGTGTCTTTATTTATTTTACCATAGCATAAATATTAATTTAGCTGAGACTGTAAACTATTGTGCAATAAGAAATCCATCAAAGTGAGAGCAGCATCTGGTTTTATTGTTGTTAAAA
TTACATTTTTATCACATGATAATGTAGCTCAAGTGCAGCTTAAATTAAAAACTGCCCTGCACTAATCACAATATTTGTGAAACAGAGACAATGGATTACACATGTCAAGCAAGTCTCAGCA
TGTTGATTTTTATACTGTGTAAAACTGAATGGGATTGACACATGGAAAAATGTAAAAAGAGAAGAGGAAAATAGTTCTCAGTAATGTGTAGAAAATGGACAGAAACATTCAAAATCACT
GCTATAGTCCTTTAAGTTGCAAAATGGCATGGGATTACTAAAGAAAAAAAACACTGTGTGCAGAAAAAAGAAAAAAATGTCCTGCTTTACTGTTAGTGAGTGCAATTGTGTGACTTTGT
ATCATTTGCACCTGCTTTATTGCTTGCTTAACTACTTAGTTGTTACTGTTATGTTGTAACAGATTTTTGTGATGCTGCAGCTTATATTTTAGCAGTGTGTGAATTGGAGATTAGTGGCTACTT
CGGATATAACTAGGGAACCTACGTGCAGCTGATGCAACCGGCCCCTGACACCTTTGCATCCTCACTTGGAATTAGGCACAGGTCAATGGGGGTCTTATTCCCACTGTGTATCAGCACACC
CAGTCTGACACACATAACTGTACACAAAACAATGACTTTCTCTGCCTCTTATACATACACACAAGCAAACACACAATATTTCCTTCCTCTCTTACACACATACAGTACATATATGACAAAGT
TATCCTAAAGAATACACAAACATTCTCTTTCTGGAATCTCCAAATCTAAGACTGAAGGCCAATGCCACCCTTAATATGGGAGTGTATTATATGGCTAGGACACAGGAACGTTGCTTGTTG
CTTCATTTCTGTTCAAGCCTGATCCCTCTACTTCTGTCTTTGAATGTTAAATAGTTGTTCTTCAAGCCCTGCACCACTGCCTCAACTGCTTCAGTGCTTGTAGGATTTGCTTGCCCGCTGCCT
GTTTTCCCCCCTCTACTCCTGTTACTCCAGATCAACATGGTTGGACCACATCTTCCAAGTCACTCTCCTTCAGTGAACTGTACAGTTGTTGTAATGCAGCAACTTTTGTGTATTTGCATATT[
CATGCACAGACACACACACACACACAGACACATGCACATGCACATACACATACACATACACTACACACACACACACACATTTT]TTGTTAGGTTCAACTTCAGCTGAGCTTAGGCTGCTTC
AGCCAGAGTGTCTCTTCTGATTGCTCATTGTCGACAGCTCTGTCATCCCTTGCATCCGTTCTATGCGTCACATTGTGAGGACATCTGACAGGTTAGCAAAAAGTGAGAAATATGTTGTAGT
GGATCATAATAAGACAAGATTAAGAGGGCAAAAGAGGAGTCAGAAAAAGCCAGAAAAAAGCAGGCATTGTAGTGTAGTGTGCAGCTCCAACATAGATTGGGCAAGTCATTCCTGTTTT
GTACAAACAGTGGTATTCTAGTTATTCCTGTGTCAGCCATTAGTACGAACTGAGCAACTTGGGTCAATTAAAACCACTTAAAAAAAAATATGTGCAGTGCAGTTCAGCAGCAGTGGAAA
GATGTTGATGATTGACTTTGGGGGGGGGGGATCTCATGAGTTAATGGAGGAGAAACACACTCTTAACATTGTTTATGGTGCACTATTGAGAGGTAACAACATGAAAGGCGAACCCAGA
AAGCAAGCCAAAATCATAGCTCCCAAGACCTTTAAATTATACAGTTGCCTAGTTGCTGCACATCAACATCAGAAACAAGACTGCTGGGAAGTGCCATATTGAAGAAACTTCTACAAGCTG
CAGTGACCCCAGGGTGAACTGATCAACTTCATTTGGCCCTGATGTTATGAAACCCAGATCTGGCATGGTAGAGGAAGGTTTCTGCAAGCAGTGAATACTGGAACAGCATCAACGTGATG
AAGTGTCATAAAATCTCACCTCAGTGTTCCTTTTTTTAGCCCATCGTATTACCAGTCACAGTAGGGGTATATAGGTGCTGCTATCAGGCTCAATCCCTCCCATGCACATGTGTCGCCCCCCC
CCCCAGCCCCGTCCCGTCCTTCAGGTGTACTTCTGTGCTGTCAGATCACCAAGTGGCTGTTTGCATGTTATAAGCAAAGGAAACAGATACTATATAGATACTATATATACATAGATATGTT
TTTCCACTGGGAAATGCGTATTGGAATACATATACCTCTGATACACATTCTCAAAGACAATACACACAATGAAAGACCCACACTCATCCCATACACACATAAATACATACAAACATATACT
CTATTGCGAACAGTAATTTGTGGAAAAACCTGAAAGAATCACATTTTTTATCCTGTTGAAAATGAATTTAATCTTCTAGATATACATAATGCTATTGTGTTATGCTAAGAAATGCCTTTTTT
CTGTTTTTACCCA -3’

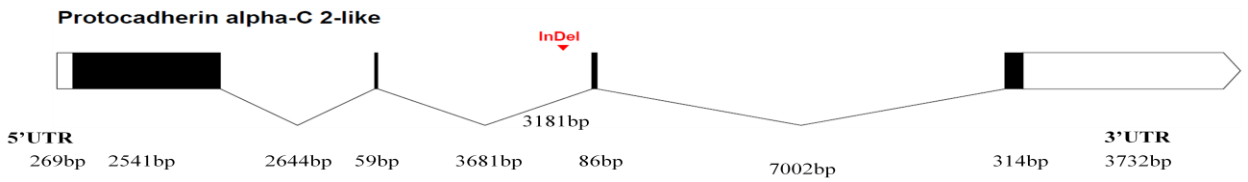

Supplementary Fig. S2 Schematic representation of *protocadherin alpha-C 2-like*; six bp InDel was located in 3181 bp in the 2th intron

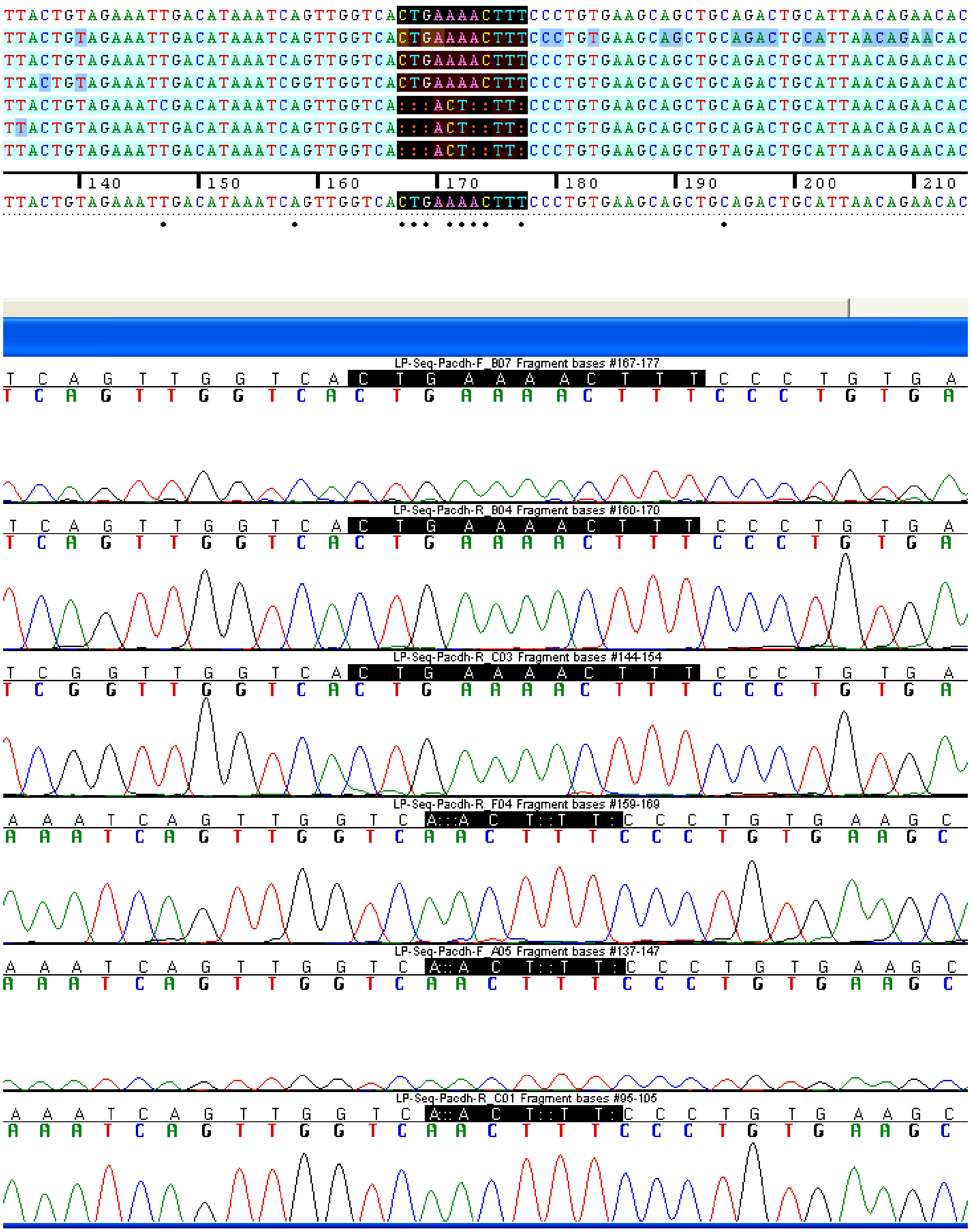

Supplementary Fig. S3 The six bp InDel in the 2th intron of *protocadherin alpha-C 2-like* of parents for mapping population
